# Enterococcal meningitis caused by *Enterococcus casseliflavus*. First case report

**DOI:** 10.1186/1471-2334-5-3

**Published:** 2005-01-14

**Authors:** Chiara Iaria, Giovanna Stassi, Gaetano Bruno Costa, Rita Di Leo, Antonio Toscano, Antonio Cascio

**Affiliations:** 1 AILMI (Associazione Italiana per la Lotta contro le Malattie Infettive), Università di Messina, Italy; 2 Servizio di Microbiologia, Università di Messina, Italy; 3 Dipartimento di Neuroscienze, Università di Messina, Italy; 4 Scuola di Specializzazione in Malattie Infettive, Dipartimento di Patologia Umana, Università di Messina, Italy

## Abstract

**Background:**

Enterococcal meningitis is an uncommon disease usually caused by *Enterococcus faecalis *and *Enterococcus faecium *and is associated with a high mortality rate. *Enterococcus casseliflavus *has been implicated in a wide variety of infections in humans, but never in meningitis.

**Case presentation:**

A 77-year-old Italian female presented for evaluation of fever, stupor, diarrhea and vomiting of 3 days duration. There was no history of head injury nor of previous surgical procedures. She had been suffering from rheumatoid arthritis for 30 years, for which she was being treated with steroids and methotrexate. On admission, she was febrile, alert but not oriented to time and place. Her neck was stiff, and she had a positive Kernig's sign. The patient's cerebrospinal fluid was opalescent with a glucose concentration of 14 mg/dl, a protein level of 472 mg/dl, and a white cell count of 200/μL with 95% polymorphonuclear leukocytes and 5% lymphocytes. Gram staining of CSF revealed no organisms, culture yielded *E. casseliflavus*. The patient was successfully treated with meropenem and ampicillin-sulbactam.

**Conclusions:**

*E. casseliflavus *can be inserted among the etiologic agents of meningitis. Awareness of infection of central nervous system with *Enterococcus *species that possess an intrinsic vancomycin resistance should be increased.

## Background

Enterococcal meningitis is an uncommon disease accounting for only 0.3% to 4% of cases of bacterial meningitis which is nevertheless associated with a high mortality rate. It has been described most frequently in patients with neurosurgical conditions (i.e. head trauma, shunt devices, or cerebrospinal fluid leakage), although it can also occur as a "spontaneous" infection complicating remote enterococcal infections such as endocarditis or pyelonephritis [1].

*Enterococcus faecalis *and *Enterococcus faecium *are the two species most frequently isolated during the course of meningitis (76%–90% and 9–22% respectively). *Enterococcus casseliflavus*, first considered as a subspecies of *E. faecium*, is a motile enterococcus that produces a yellow pigment in agar and often has a VanC phenotype determining an intrinsic low level resistance to vancomycin. It has been implicated in a wide variety of infections in humans, especially immunocompromised hosts, but to the best of our knowledge it has never been associated to meningitis [2-5].

We describe here a case of enterococcal meningitis caused by *E. casseliflavus *that was believed to originate from the gut in an old patient with bowel erosions.

## Case presentation

A 77-year-old Italian female presented for evaluation of fever, stupor, diarrhea and vomiting of 3 days duration. She had no urinary symptoms. There was no history of head injury nor of previous surgical procedures. She had been suffering from rheumatoid arthritis for 30 years, for which she was being treated with steroids and methotrexate; other medical problems were insulin-dependent diabetes and moderate renal failure.

On admission, she was febrile (temperature, 38.0°C), alert but not oriented to time and place. Her neck was stiff, and she had a positive Kernig's sign. A CT brain scan showed an increase in subarachnoid space and in the volume of the ventricular system.

Laboratory examinations revealed a white blood cell count of 15,100/μL with 70% neutrophils and 23% lymphocytes. Results of urinalysis were unremarkable. Cultures of blood and urine were drawn and subsequently resulted negative.

The patient's cerebrospinal fluid (CSF) was opalescent with a glucose concentration of 14 mg/dl, a protein level of 472 mg/dl, and a white cell count of 200/μL with 95% polymorphonuclear leukocytes and 5% lymphocytes. Gram staining of CSF revealed no organisms. Pending the culture results the patient was empirically treated with intravenous meropenem, cotrimoxazole, acyclovir and dexamethasone.

Culture of CSF yielded *E. casseliflavus *that was identified using the Vitek-2 system (bioMérieux-Vitek) on the basis of 6.5% NaCl tolerance, bile-esculin hydrolysis, and growth rate at 45°C, arginine hydrolysis, methyl-a-D-glucopyranoside testing, and acid production from ribose, motility testing, and yellow pigmentation testing.

The isolate was sensitive to penicillin, ampicillin, ampicillin-sulbactam, imipenem, teicoplanin, tetracyclines and linezolid; it exhibited intermediate sensitivity to vancomycin (MIC, ≥8 μg/mL), trimethoprim-sulfamethoxazole (MIC, ≥10 μg/mL), levofloxacin (MIC, 4 μg/mL), norfloxacin (MIC, 8 μg/mL), ciprofloxacin (MIC, 2 μg/mL) and quinupristin-dalfopristin (MIC, 2 μg/mL); it was resistant to clindamycin (MIC, 4 μg/mL) and showed high resistance to gentamicin, streptomycin and kanamycin (MIC, ≥2000 μg/mL).

The patient became afebrile 48 hours after the beginning of antibiotic therapy with rapid improvement of her mental status and disappearance of meningeal signs (within 36 hours).

Once the organism was identified (4 days later), trimethoprim-sulfamethoxazole and acyclovir were discontinued and ampicillin-sulbactam (3 g every 6 hours) was added. After 2 weeks of antibiotic therapy the patient was discharged in good health with sterilization of the CSF culture. An echocardiogram revealed no vegetations whereas a colonoscopy examination showed two ulcerative lesions associated with two polyps, oedema and multiple punctuate erosions.

Enterococcal infections of the central nervous system are quite rare and according to a MEDLINE search of the English literature only three cases of CNS infection by a motile *Enterococcus *identified as *E. gallinarum *have been previously documented; they occurred in patients with ventriculoperitoneal shunts for hydrocephalus [6,7]. *E. casseliflavus *and *E. gallinarum *are responsible for 1–2% of all enterococcal infections and are characterized by the fact that they possess intrinsic low-level vancomycin resistance [8]. The VanC-1 ligase is specific for *E. gallinarum*, and the VanC-2/3 ligase is specific for *E. casseliflavus *[9]. Organisms with resistance to VanC remain susceptible to teicoplanin. This naturally occurring vancomycin resistance has not been shown to be transferable, and the related genes are chromosomally encoded in the members of these species [4,9,10].

Despite the intrinsic low-level vancomycin resistance exhibited by *E. casseliflavus *it is important to remember that most strains are susceptible to penicillin and ampicillin. Combination therapy of ampicillin with an aminoglycoside such as gentamicin or streptomycin is considered the standard therapy of enterococcal meningitis due to ampicillin-susceptible strains [1]. Meropenem is not superior to ampicillin for therapy of enterococcal infections and most species of *E. casseliflavus *are beta-lactamase negative. There was not therefore a clear indication for the use of combination therapy with meropenem and a beta-lactamase inhibitor in the case reported.

Our patient had gastrointestinal signs and symptoms, and colonoscopy revealed multiple erosions, which probably were the portal of entry of *E. casseliflavus*.

In fact, enterococcal meningitis may appear as a complication of diverse gastrointestinal diseases such as enterocolitis, peritonitis, abdominal surgery, or bowel carcinoma. A case of bowel erosions as the portal of entry of enterococcal meningitis has been previously reported [11].

Several studies have demonstrated that *E. gallinarum *and *E. casseliflavus *colonize the gastrointestinal tracts of both hospitalized individuals and nonhospitalized healthy ones [8,12,13]. Therapy with various antimicrobial agents, including cephalosporins and vancomycin, may play a role in increasing colonization with these organisms. Edlund et al. reported a significant increase in the emergence of *E. gallinarum *and *E. casseliflavus *in healthy subjects who were administered oral vancomycin [14]. Our patient was not taking any antimicrobials before hospital admission. *E. gallinarum *and *E. casseliflavus/flavescens *are part of the normal stool flora of the general population; this has perhaps impacted the ability of researchers to detect specific risk factors [8].

Although our patient had significant underlying conditions this case of meningitis was mild and had most of the typical features of "spontaneous" enterococcal meningitis (community-acquired infection, severe underlying diseases, and immunodepression).

The clinical significance of the enterococci that are intrinsically resistant to vancomycin has not been fully established yet. Infection with *E. gallinarum *and *E. casseliflavus *has been associated with high mortality, but it is difficult to attribute their mortality directly to infection or to the underlying conditions of the patient.

## Conclusions

This case is the first report of *E. casseliflavus *meningitis and it is the fourth documented so far with a motile *Enterococcus *species. Awareness of infection of central nervous system with *Enterococcus *species that possess an intrinsic vancomycin resistance should be increased.

## Competing interests

The author(s) declare that they have no competing interests.

## Authors' contributions

IC and RDL carried out the clinical study of the patient and conceived of the study. GS and GBC carried out the microbiologic studies. AT and AC carried out the clinical study of the patient, conceived of the study and drafted the manuscript. All authors read and approved the final manuscript.

## Pre-publication history

The pre-publication history for this paper can be accessed here:


